# The breadth of animacy in memory: New evidence from prospective memory

**DOI:** 10.3758/s13423-023-02406-y

**Published:** 2023-11-27

**Authors:** Sara B. Félix, Marie Poirier, James S. Nairne, Josefa N. S. Pandeirada

**Affiliations:** 1 William James Center for Research, Department of Education and Psychology, University of Aveiro, Campus Universitário de Santiago, 3810-193 Aveiro, Portugal; 2 Department of Psychology, School of Health and Psychological Sciences, City, University of London, London, UK; 3 Department of Psychological Sciences, Purdue University, West Lafayette, USA

**Keywords:** Animacy effect, Adaptive memory, Prospective memory

## Abstract

Studies using retrospective memory tasks have revealed that animates/living beings are better remembered than are inanimates/nonliving things (the animacy effect). However, considering that memory is foremost future oriented, we hypothesized that the animacy effect would also occur in prospective memory (i.e., memory for future intentions). Using standard prospective memory (PM) procedures, we explored this hypothesis by manipulating the animacy status of the PM targets. Study 1a reports data collected from an American sample; these results were then replicated with a Portuguese sample (Study 1b). Study 2 employed a new procedure, and data were collected from a broader English-speaking sample. In these three studies, animate (vs. inanimate) targets consistently led to a better PM performance, revealing, for the first time, that the animacy advantage extends to PM. These results strengthen the adaptive approach to memory and stress the need to consider animacy as an important variable in memory studies.

**Supplementary Information:**

The online version contains supplementary material available at 10.3758/s13423-023-02406-y.

Evolutionary psychology postulates that human cognition (e.g., memory) evolved to help solve adaptive problems, such as finding food and shelter (Cosmides & Tooby, 1992; Nairne et al., 2017). Thus, researchers have hypothesized that there is a memory tuning for fitness-relevant information (i.e., information that enhances our odds of survival and/or reproduction). An example of fitness-relevant information relates to animacy, as animates are fitness-relevant in many ways (e.g., they can represent predators, prey, sexual mates, among others; Nairne et al., 2017). Animacy has been operationalized in many ways (e.g., as a synonym of *agency* and *livingness*; for an overview, see Félix et al., 2023). According to VanArsdall and Blunt (2022), the *livingness* construct loads highly onto the *animacy* factor; thus, animacy will be conceived here as *livingness* (as in most memory research; e.g., Nairne et al., 2013), or the distinction between living beings (e.g., humans and nonhuman animals) and nonliving things (e.g., objects). Indeed, people tend to remember animates better than inanimates, a phenomenon called the “animacy effect.”

Since the first report showing that animacy is one of the best predictors of free recall (Nairne et al., 2013), the animacy effect has proven to be robust in retrospective memory tasks (i.e., memory for past events); it has been reported using a variety of procedures, types of to-be-encoded stimuli, and in different languages (e.g., free recall, with French words and pictures as the to-be-remembered stimuli: Bonin et al., 2014; metamemory/judgements of learning in English: DeYoung & Serra, 2021; implicit memory in Spanish: Laurino & Kaczer, 2019; working memory: Daley et al., 2020; directed forgetting in English: Murphy & Castel, 2022). There are, however, some circumstances in which evidence is less clear (e.g., recognition: Bonin et al., 2014; Leding, 2020; cued recall: Popp & Serra, 2016; but see VanArsdall et al., 2015).

Allied with the importance of retrieving information from the past, some authors have suggested that our memory is foremost future oriented: One of our memory’s main function is to store information from the past in order to help solve problems in the present and predict/get prepared for future events, which is crucial for survival (Ingvar, 1985; Klein, 2013; Nairne & Pandeirada, 2008; Schacter et al., 2007). This relates directly with prospective memory (PM), which is the memory for upcoming plans, events, actions or intentions to be performed in the future (Einstein & McDaniel, 1990). Everyday examples of PM tasks are to remember to deliver a message to a friend when he/she is encountered, to take the pills after lunch, or to remember to return books at the library the following day. Importantly, prospective memory tasks mostly involve other people (i.e., animates), and PM successes or failures can impact individuals themselves, as well as their relations with others, thus having clear adaptive consequences. For instance, there would be a benefit conferred by remembering to avoid cheaters in future encounters; likewise, remembering to maintain positive interactions with cooperators in the future would also be advantageous (Schaper et al., 2022). Failure to remember to pick up the kids from school or to remove a clamp from the patient’s abdomen (Brandimonte & Ferrante, 2008; Dembitzer & Lai, 2003) also illustrate this point. In the present work, we aimed to combine these two adaptive elements—the animacy variable and prospective memory—and explore whether animates also confer an advantage in PM performance.

Prospective memory has been studied in the laboratory using several types of tasks (see Kvavilashvili & Ellis, 1996, for further details). Our work focused on event-based tasks, in which the moment to perform the intention is signaled by the presence of a specific event–the PM target (e.g., whenever you see John [target], give him a message [PM response]). Laboratory PM studies usually employ a dual-task paradigm, that is, the PM response occurs while another task is ongoing. In a typical procedure, participants first respond to the ongoing task (e.g., a lexical decision task), which provides a baseline to their performance on that task alone; these are called the *baseline trials*. Then, the PM instructions are presented: Participants are tasked to provide an alternative response (the PM response) whenever specific targets appear (e.g., press F1 whenever the syllable “TOR” appears; McDaniel & Einstein, 2000) while performing the ongoing task. The trials involving the target words are the *target trials*, whereas those regarding the ongoing task are now named the *filler trials*. Although animacy has never been systematically manipulated or analyzed in PM (i.e., was not an independent variable in such studies), animates (e.g., animals) and/or inanimates (e.g., clothing, furniture) have been used as PM targets in event-based PM tasks (e.g., Chen et al., 2014; Marsh et al., 2009). For example, in one of the seminal works on PM (Einstein et al., 2005), among the targets we find an animate (*tortoise*), an inanimate (*dormitory*) and an ambiguous word (*tornado*; although classified as an inanimate, it can be perceived as an animate, due to the sense of self-propelled motion and agency; Lowder & Gordon, 2015). Other studies (e.g., Moyes et al., 2019) have asked participants to provide the PM response whenever targets from a specific category were presented, including categories of animate items (e.g., four-footed animals), ambiguous words (e.g., flowers, fruits), and inanimate words (e.g., metals); once again, no information was provided regarding the influence of animacy on PM performance.

Here, in a series of three studies, we explored the animacy effect in PM. We expected higher PM performance when the target was an animate (e.g., “horse”), comparatively to when it was inanimate (e.g., “shirt”). No strong predictions were made about the animacy effect for the baseline and filler trials. However, as it has been suggested that animates capture attention more automatically than inanimates (e.g., Bugaiska et al., 2019), the former would capture participants’ attention and divert it from the ongoing task. As a result, it would be reasonable to anticipate a decline in the ongoing task when trials (baseline and filler trials) presented animate words compared with inanimate ones.

Again, our main interest was to explore the animacy effect on the PM target trials. Three factors explain the prediction of an animacy advantage in PM: First, both PM and the animacy variable entail adaptive value. Second, people tend to judge animates as more memorable than inanimates for a future memory test (e.g., DeYoung & Serra, 2021); considering that there is a correlation between those judgements of learning and the actual PM performance (Schnitzspahn et al., 2011), it is conceavable that an interplay among metacognitive judgments, animacy, and prospective memory may occur leading to an animacy advantage in PM. Third, most theories on PM were developed based on knowledge about retrospective memory functioning (McDaniel & Einstein, 2000). Consistently, several variables known to influence retrospective memory also influence prospective memory. For instance, emotional words/targets, as compared with neutral ones, enhance both retrospective (e.g., Dewhurst & Parry, 2000) and PM performance (Hostler et al., 2018; May et al., 2015). Also, Smith (2003) found that distinctive words (i.e., targets with a distinctive orthography, such as sphinx), as compared with common orthography words, improved both prospective memory and recognition performance. Given that animates (vs. inanimates) are better remembered in retrospective memory tasks (e.g., free recall: Nairne et al., 2013; working memory: Daley et al., 2020), one could expect the same advantage to occur in PM (e.g., see the relation between working memory and PM; Brewer et al., 2010).

## Study 1a

This study used a well-known PM procedure: while performing an ongoing color-matching task (Smith & Hunt, 2014), participants were required to provide an alternative response (PM response) whenever either of two predefined target words (one animate and one inanimate) appeared. Across studies, we included a baseline phase (color-matching task only) and a PM phase (ongoing color-matching task with an embedded PM task). Of particular interest will be the results regarding the PM performance.

### Method

#### Participants

Using G*Power (Version 3.1.9.7; Faul et al., 2007), we predetermined that a sample size of 109 participants was needed to obtain a small-medium effect size, *dz* = 0.35, with *α* = .05 and power = .95. A total of 351 Purdue University undergraduate students participated in exchange for course credits. From those, 175 participants were excluded from the analyses: 51 participants were non-English native speakers; 54 did not provide any PM response; 42 reported having cheated and/or not paid attention to the study or had extremely long survey durations (>7.4 hours; which may reveal low engagement with the task and/or low attention, or a start-and-stop behavior throughout the task despite the instruction to respond to the task in just one sitting); 13 participants did not recognize one (or both) target word(s) and did not provide any PM response to those target trials; 10 had more than 50% missing responses to the ongoing task; four participants had low performance on the filler trials/ongoing task (<Grand Mean – 3 *SD*); and another participant was underaged. See Supplemental Materials for additional information about the sample.

The final sample was composed of 176 participants (31.3% females and 68.8% males; mean age = 19.43 years, *SD* = 1.17). They were all English native speakers or bi-/multilingual [being proficient in English and other(s) language(s)]. Forty to 46 participants were allocated to each version of the task (see Procedure).

#### Material

Animate and inanimate words were selected from VanArsdall (2016), which reports animacy norms for a large set of words. Sixteen words were selected for the baseline phase. For the PM phase, a new set of 24 filler and two pairs of target words were selected to increase the generalizability of the results. Two additional words were selected for the practice trials. In all cases, half the words were animate and the other half were inanimate (see Supplemental Materials). The animate and inanimate words were matched along a number of relevant mnemonic variables (see Table 1).

**Table 1:** Characterization of the animate and inanimate words used in Studies 1a, 1b, and 2

**Study 1a and Study 2**									
	Baseline words (*n* = 16)			Filler words (*n* = 24)			Target words (*n* = 4)		
	Animates	Inanimates	*p*	Animates	Inanimates	*p*	Animate	Inanimate	*p*
Anim.^a^	6.83 (0.10)	1.07 (0.04)	***	6.84 (0.15)	1.01 (0.02)	***	6.78 (0.14)	1.02 (0.03)	**
AoA ^b^	3.00 (0.48)	3.18 (0.24)	.408	2.95 (0.56)	3.33 (0.75)	.176	4.70 (1.83)	2.72 (0.22)	.366
Arou.^c^	4.85 (0.93)	3.96 (0.98)	.085	4.44 (0.53)	4.26 (0.70)	.506	5.42 (0.82)	4.11 (0.96)	.283
Conc. ^d^	5.96 (2.92)	5.94 (2.87)	.896	5.94 (0.31)	5.95 (0.18)	.944	5.73 (0.21)	6.08 (0.23)	.263
Dom.^c^	5.44 (0.99)	5.11 (0.47)	.412	5.38 (0.55)	5.28 (0.40)	.618	5.43 (0.83)	4.61 (0.24)	.385
Fam.^d^	5.37 (0.60)	5.63 (0.45)	.332	5.42 (0.48)	5.60 (0.39)	.306	5.36 (0.01)	5.71 (0.29)	.341
Freq.^e^	100.88 (93.35)	54.63 (58.74)	.259	41.33 (63.42)	27.83 (20.34)	.495	24.00 (9.90)	65.00 (15.56)	.108
Img.^d^	6.07 (1.86)	5.97 (2.37)	.379	5.98 (0.18)	5.95 (0.15)	.637	5.84 (0.47)	6.03 (0.23)	.673
Length	4.50 (1.07)	5.50 (1.93)	.226	4.75 (1.48)	4.75 (0.87)	>.99	5.50 (0.71)	5.50 (0.71)	.999
Val.^c^	6.26 (0.98)	5.94 (0.98)	.468	5.85 (1.05)	5.64 (0.85)	.612	6.61 (0.75)	6.12 (0.04)	.525
**Study 1b**									
	Baseline words (*n* = 16)			Filler words (*n* = 24)			Target words (*n* = 4)		
	Animates	Inanimates	*p*	Animates	Inanimates	*p*	Animate	Inanimate	*p*
Anim.^f^	6.65 (0.12)	1.52 (0.11)	***	6.76 (0.06)	1.50 (0.08)	***	6.64 (0.10)	1.44 (0.06)	**
AoA ^g^	3.03 (1.02)	2.34 (0.62)	.130	2.54 (0.67)	2.87 (0.90)	.302	1.66 (2.34)	2.11 (0.48)	.831
Arou.^h^	4.36 (0.40)	3.89 (0.69)	.120	4.15 (0.39)	4.05 (0.44)	.586	2.85 (4.03)	3.27 (NA)	NA
Conc.^i^	6.38 (0.21)	6.36 (0.40)	.890	6.42 (0.38)	6.46 (0.30)	.785	6.11 (0.05)	6.71 (0.01)	.033
Dom.^h^	5.08 (0.58)	5.13 (0.61)	.868	5.17 (0.45)	5.11 (0.45)	.742	1.89 (2.67)	4.45 (NA)	NA
Freq.^i^	21.15 (23.35)	58.87 (82.03)	.246	21.81 (24.13)	24.49 (39.83)	.844	24.40 (33.94)	17.82 (5.72)	.830
Img.^i^	5.64 (0.22)	5.84 (0.32)	.155	6.05 (0.30)	6.06 (0.27)	.972	5.24 (0.56)	5.96 (0.29)	.284
Length	5.63 (1.19)	5.50 (1.31)	.844	5.50 (1.57)	6.00 (1.28)	.401	5.50 (0.71)	6.50 (0.71)	.293
S.Freq. ^i^	4.50 (1.10)	5.15 (1.19)	.275	4.69 (0.93)	4.91 (1.16)	.623	3.82 (1.15)	5.36 (1.19)	.317
Val.^h^	5.54 (0.87)	5.80 (0.55)	.495	6.04 (1.00)	5.60 (0.70)	.258	2.01 (2.84)	5.55 (NA)	NA

*Note.* Mean values presented, with standard deviations in parentheses; *n* = Number of words (containing half animate and half inanimate); NA = Not Available; *p* = *p* value obtained by independent *t* tests (animate vs. inanimate); Baseline words = Words used in the baseline trials; Filler words = Filler words used in the PM phase; Target words = Words used in the target trials in the PM phase.

Anim. = Animacy; AoA = Age of Acquisition; Arou. = Arousal; Conc. = Concreteness; Dom. = Dominance; Val. = Emotional Valence; Fam. = Familiarity; Freq. = Written frequency; Img. = Imageability; Length = number of letters of the words; S. Freq. = Subjective Frequency.

**Word data for Studies 1a and 2 retrieved from**: ^a^ VanArsdall and Bunt (2022) [7-point scale]; ^b^ Cortese and Khanna (2008) and Schock et al. (2012) [7-point scale]; ^c^ Bradley and Lang (1999) [9-point SAM scale]; ^d^ MRC database (Wilson, 1988) [transformed into a 7-point scale]; ^e^ Kučera and Francis (1967) as available in the MRC database (Wilson, 1988). Baseline words: The word “jug” missed values for concreteness and imageability; No age of acquisition information was available for the words “umbrella” and “horse”. Filler words: No data on emotional valence, arousal and dominance were available for the words “monkey” and “jacket.” Target words: Data on emotional valence, arousal and dominance for the word “phone,” and data on AoA for the word “dancer” were retrieved from VanArsdall (2016).

**Word data for Study 1b retrieved from**: ^f^ Félix et al. (2020) [7-point scale]; ^g^ Average data from Cameirão and Vicente (2010) and Leitão et al. (2010) [transformed to a 7-point rating scale]; ^h^ Soares et al. (2012) [9-point SAM scale]; ^i^ Soares et al. (2017) [7-point scale]. Target words: Data on emotional valence, arousal, and dominance for the word “camisa [shirt]” were not available in the few existing European Portuguese databases that also contain a reduced number of words.

***p value < .001; **p value < .01

#### Procedure

Data were collected online using Qualtrics, in sessions lasting, on average, 25 minutes. The procedure was similar to that used by Smith and Hunt (2014), except that words were presented in a fixed order to every participant (their order was pseudorandomized, ensuring that each quarter of the list had a balanced number of animates and inanimates), and we used fewer trials, aiming for a shorter task; however, we used the same proportion of target trials during the PM phase as in their work (~8%). There were two predetermined presentation orders in the PM phase to ensure that, in each position of the list, an animate and an inanimate item was presented an equal number of times across participants. We also used one out of two sets of PM targets (*dancer* and *bottle* / *nurse* and *phone*) in each of these versions; their presentation order was predetermined within the list of items and counterbalanced across participants such that, animate and inanimate targets appeared equally in each target position. Therefore, there were four versions of the task to which participants were randomly assigned.

**Fig. 1:**
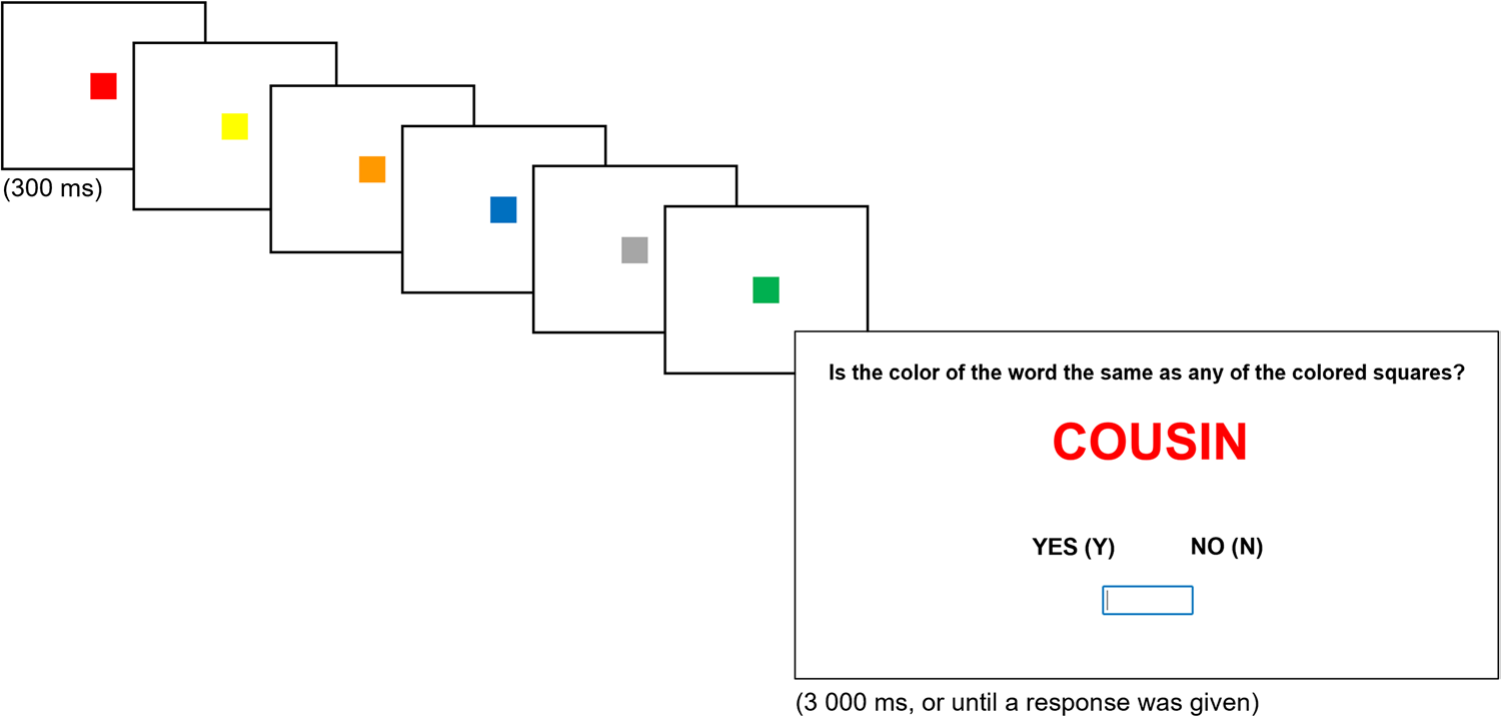
Example of a match-trial, and representation of the presentation times of each stimulus (Study 1a). *Note*. In this example, the colors of the squares are presented in the following order: red, yellow, orange, blue, gray, and green. The word “COUSIN” is in a red-colored font; the correct response for this trial would be Y (yes). (Color figure online)

After consenting to participate, participants received the instructions for the ongoing task. Specifically, they were told that six colored squares would be presented, one at a time, each one in a different color (red, yellow, blue, green, pink, orange, or gray). Then, a word would be presented in a colored font. Participants had to decide whether the color font of the word matched the color of any of the just-presented squares by pressing the Y (yes) or the N (no) keys (see Fig. 1 for an illustration of the procedure). Participants started by responding to two practice trials to get familiar with the task. Then, they were reminded of the ongoing task instructions and performed the baseline phase (16 trials; color-match ongoing task only). Throughout the experiment, half of the trials (animate and inanimate) were match-trials (i.e., the color font matched the color of a square), and the other half were nonmatch-trials.

After the baseline phase, participants read the PM task instructions which informed them that they were to memorize two new words (an animate and an inanimate word; PM targets). Also, participants were told they would need to press the SPACEBAR (PM response), instead of Y/N, whenever any of these words appeared during the color-matching task. The target words were then displayed simultaneously for one minute. A 2-min distractor task followed (a 3D mental rotation task; Ganis & Kievit, 2015) to prevent participants from rehearsing the PM instructions.

Next, the PM phase began without further reminders of the PM instructions. In this phase, participants were presented with two target and 24 filler words (half animate, half inanimate). To increase the number of PM target trials during the task, each target was presented twice. The filler words were also repeated to prevent target words from becoming distinctive (Smith, 2003); they appeared in a different color each time, once in a match and once in a nonmatch-trial, totalizing 48 filler trials. The PM target trials were presented in the 11th, 24th, 36th, and 51st trials/positions of the list.

Upon completion of the PM phase, participants were asked to recall the instructions they received for the task. They also performed a target recognition test: participants were presented with a short series of six words, one at a time, and asked whether each word corresponded to a PM target (yes/no); both the targets presented in the task and four lures (half animates and half inanimates) from the PM phase were presented. A color-naming task followed. These words and colors were presented one at a time, in a random order for each participant. Finally, participants provided sociodemographic information (age, gender, and native language); also, they responded to “honesty questions” regarding whether they paid attention and answered honestly to the task (Rouse, 2015). They were asked to provide optional feedback regarding the study, were thanked, and debriefed.

### Data analyses

Data from all the three studies were analyzed using SPSS 28 (IBM Corp, 2021). The main dependent variable was the proportion of correct responses (i.e., press the correct key—Y, N, or SPACEBAR—in match, nonmatch and target trials, respectively). We conducted a 2 (Animacy: animates vs. inanimates) × 3 (Type of Trial: baseline vs. filler vs. target) repeated-measures analysis of variance (ANOVA; we report the Greenhouse–Geisser corrected data, as the sphericity assumption was violated in all analyses). We used additional paired-sample *t* tests with Bonferroni corrections (*p* < .05/3) to clarify some results. Supplemental Materials present additional analyses, namely: on response times, excluded participants, false alarms, as well as data confirming that the overall results here reported hold when we consider the different sets of PM targets used across experiments.

### Results

**Fig. 2:**
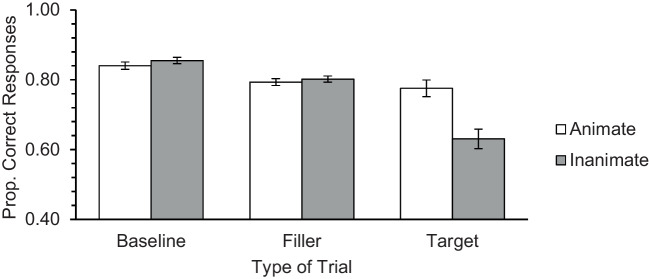
Mean performance obtained in baseline, filler (ongoing task) and target trials (PM Task), in Study 1a. Error bars represent standard errors of the mean

Results are presented in Fig. 2. A significant Animacy main effect was observed, *F*(1, 175) = 12.48, *p* = .001, η_p_^2^ = .067, revealing that performance was better for the animate than the inanimate stimuli. The main effect of Type of Trial also reached significance, *F*(1.29, 224.89) = 38.87, *p* < .001, η_p_^2^ = .166. A significant Animacy × Type of Trial interaction was also obtained, *F*(1.20, 209.31) = 18.36, *p* < .001, η_p_^2^ = .095. The follow-up paired *t* tests performed on each type of trial revealed that the animacy advantage was significant only on the target trials, *t*(175) = 4.39, *p* < .001, *d*_z_ = 0.33. No animacy advantage was obtained for the baseline, *t*(175) = −1.27, *p* = .206, or filler trials, *t*(175) = −1.08, *p* = .281.

## Study 1b

Study 1a was the first study reporting an animacy advantage in PM: Animate targets elicited better PM performance than the inanimate targets did. As with any first discovery, more empirical evidence is needed for the effect to be considered reliable. Study 1b aimed to replicate the findings from Study 1a with a group of participants from another country and language. The same procedure was employed, except that participants in this study were Portuguese, and a new set of stimuli was selected from existing norms for European Portuguese.

### Method

#### Participants

Using G*Power (Version 3.1.9.7), *N* was set as 76 participants (*α* = .05, power = .85) to achieve a small to medium effect size (*d*_z_ = 0.35). A convenience sample of 163 university students participated in exchange for course credits or a prize draw. From those, 85 participants were excluded from the data analysis: 38 did not provide any PM response; 18 were nonnaïve as they took part in other PM studies from our lab; 14 participants stated not having paid attention, having cheated during the experiment, or had extremely long survey durations (>3.7 hours); 10 were non-European Portuguese native speakers or did not reveal their native language; two did not recognize one target and did not provide PM responses to those target trials; one gave no responses to more than 50% of the ongoing task trials; another was excluded due to a technical problem with the stimuli presentation; and another was underaged. Additional information is available in the Supplemental Materials.

The final sample was composed of 78 European Portuguese native speakers (mean age = 21.60 years, *SD* = 4.39; one participant was a Portuguese–English bilingual). Of those, 82.1% identified themselves as females, 16.7% as males, and 1.3% preferred not to reveal their gender. Each version of the task had 17 to 22 participants.

#### Material and procedure

The procedure was the same as in Study 1b, except that participants were asked to press S or N, for Yes [*Sim*] or No [*Não*] responses, respectively. Animate and inanimate European Portuguese words were selected from Félix et al. (2020) and matched along several variables (Table 1; selected words available as Supplemental Materials). Participants took, on average, 33 minutes to complete the task.

### Results

**Fig. 3:**
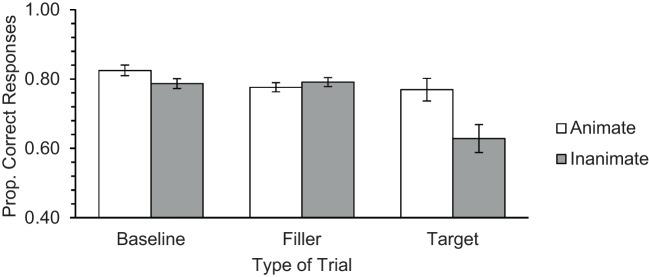
Mean performance obtained in baseline, filler (ongoing task) and target trials (PM Task), in Study 1b. Error bars represent standard errors of the mean

Results are depicted in Fig. 3. The Animacy main effect was significant, *F*(1, 77) = 10.96, *p* < .001, η_p_^2^ = .125, as was the Type of Trial main effect, *F*(1.28, 98.77) = 10.21, *p* < .001, η_p_^2^ = .117. Furthermore, the Animacy × Type of Trial interaction reached significance, *F*(1.33, 102.50) = 6.29, *p* = .002, η_p_^2^ = .076. Follow-up paired *t* tests revealed, again, a significant animacy advantage only on the target trials, *t*(77) = 2.93, *p* = .005, *d*_z_ = 0.33. No animacy effect was obtained in the filler, *t*(77) = −1.11, *p* = .270, or on the baseline trials, *t*(77) = 2.15, *p* = .035 (a nonsignificant result considering the Bonferroni correction).

## Study 2

Study 1b replicated the findings from Study 1a: The animacy effect was obtained on PM target trials. In Study 1b, a new set of stimuli was used, and the study was conducted with participants from another country and language, allowing the generalizability of the results and revealing that the effect is not language dependent. Looking for more evidence of the animacy effect in PM, Study 2 used a new ongoing task (a visuospatial task) as the main procedure.

### Method

#### Participants

The sample size was calculated as in Study 1b. A total of 130 participants were recruited from Testable Verified Minds (https://www.testable.org/) using the following prescreeners: age (18–40 years old), first language (English), location (USA, UK, Ireland, New Zealand, Canada, or Australia). Following the preregistered exclusion criteria, 51 participants were excluded: 22 did not perform any PM response; 16 had a low performance in the filler trials (<Grand Mean – 3 *SD*); five participants were non-English native speakers or preferred not to reveal their native language; three participants did not provide any response to 50% or more trials; another three did not recognize one of the targets and did not perform any PM response to those target trials; and, another two failed both attention checks. Additional information is available as Supplemental Materials.

Seventy-nine participants were included in the data analysis (mean age = 30.33 years, *SD* = 6.52; one participant did not reveal his/her age; 48.1% were females and 51.9% males). Seventeen to 22 participants responded to each version of the task.

#### Material and procedure

We used the same stimuli and procedure as in Study 1a, except for the ongoing task: Participants now performed a visuospatial task. Also, the distractor task was an even/odd task. Finally, the experiment presented two attention checks: one right after the practice and the other after the baseline phases (“Have you ever walked on Mars?” and “Can you fly with invisible wings?”—yes/no responses), which served to exclude inattentive participants (i.e., those who responded “yes” to both questions; VanArsdall, 2016). On average, the experiment lasted 26 minutes.

**Fig. 4:**
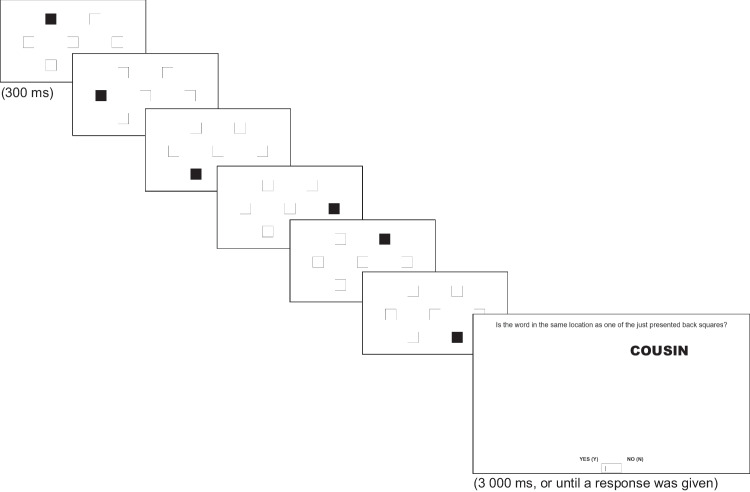
Example of a match-trial, and representation of the presentation times of each stimulus (Study 2)

In the ongoing task (Fig. 4), inspired by Costa et al. (2013), seven white squares were displayed on the screen. One at a time, six of them turned black, each one in a different location. Then, a word was presented in one of the seven possible square positions. The participants had to decide if the word’s location matched the location where a black square was displayed by pressing Y (yes) or N (no). For the PM phase, participants were instructed to press the SPACEBAR whenever any of the targets (an animate and an inanimate) was presented while performing the ongoing task.

### Results

**Fig. 5:**
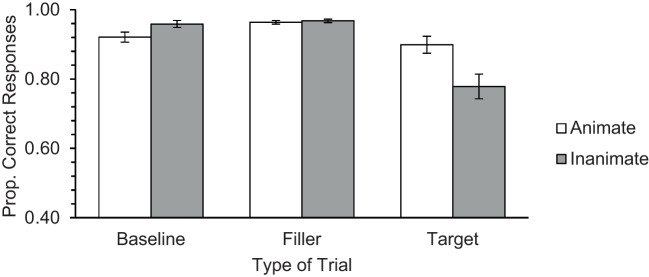
Mean performance obtained in the baseline, filler (ongoing task) and target trials (PM Task), in Study 2. Error bars represent standard errors of the mean

Results are depicted in Fig. 5. The Animacy main effect did not reach conventional levels of significance, *F*(1, 78) = 3.51, *p* = .065, η_p_^2^ = .043, but the main effect of Type of Trial was significant, *F*(1.32, 103.24) = 19.18, *p* < .001, η_p_^2^ = .197. The Animacy × Type of Trial interaction was also significant, *F*(1.22, 95.03) = 11.83, *p* < .001, η_p_^2^ = .132. This was due to a higher performance for inanimate (vs. the animate) words in the baseline trials, *t*(78) = −2.99, *p* = .004, *d*_z_ = −0.34; and, more importantly, due to a significantly higher PM performance towards animate targets, as compared with the inanimate ones, *t*(78) = 3.05, *p* = .003, *d*_z_ = 0.34. No animacy effect was obtained on the filler trials, *t*(78) = −0.70, *p* = .486.

## General discussion

The proposal that memory should be tuned to remembering animates/living beings (as compared with inanimates/nonliving things) follows from the assumption that animates typically have a high fitness-relevant value (Nairne et al., 2017). Empirical evidence of the animacy advantage exists in retrospective memory but not in prospective memory. Combining two adaptive features of memory—its tuning toward animates and its future orientation—we predicted that the animacy effect would also occur in PM.

In a series of three studies using typical PM procedures, we reported, for the first time, that PM is also sensitive to the animacy dimension, at least in the type of tasks employed here (event-based tasks; cf. Einstein & McDaniel, 2005). Indeed, PM performance was consistently better in response to animate than to inanimate targets. Most participants had better performance for the animate targets (62% in Study 1a and in Study 2; and 55% in Study 1b), a smaller percentage had better PM performance for the inanimates (20%, 26% and 21% in Studies 1a, 1b and 2, respectively), and there were around 19% ties across studies (i.e., equal performance for animates and inanimates).^1^ We should note that, in each study, we only used two different PM targets (one animate and one inanimate) to prevent a high cognitive load and, consequently, low levels of PM performance (Anderson et al., 2019), while maintaining the usual proportion of target/ongoing trials (Smith & Hunt, 2014). Still, we opted to use different sets of possible targets to increase the generalizability of our results. It is also noteworthy that the present results were obtained using different ongoing tasks and in two languages (Portuguese and English), which further reinforces the relevance and generalizability of the present findings.

Considering that knowledge about PM has been derived from retrospective memory theories, we consider how the two main accounts that have been proposed to explain the animacy effect in that context might explain the current results: the attention-based and the richness of encoding accounts. The richness of encoding account suggests that animates tend to be better recalled because they naturally lead to the generation of more ideas and/or have more features than inanimates (e.g., Meinhardt et al., 2020; Rawlinson & Kelley, 2021). Those ideas, features, or associates potentially work as retrieval cues and might improve performance (for animates) in free recall. When the existence of multiple cues is irrelevant to the task at hand, such as in cued recall, there is sometimes no animacy effect (e.g., Popp & Serra, 2016). In the case of PM, having multiple cues associated with the target could hinder the access to the PM intention memory trace (association: target–intention), thus impairing the PM performance for the animate targets (McDaniel & Einstein, 2000). In the same vein, a previous study has shown that when the PM target is paired with other words/associates in a study phase, PM performance decreases; also, the more associates are paired with the PM target, the lower the PM performance is (Cook et al., 2006). All together, these data, along with the present findings, suggest that the richness of encoding is unlikely to explain the animacy advantage reported here. As the main aim of this work was to explore, for the first time, a possible animacy advantage in PM, further studies using procedures designed specifically to disentangle the potential mechanisms are needed.

The attentional account posits that animates tend to be better recalled because they recruit attentional resources in a more automatic manner, thus requiring lower activation thresholds to be detected (e.g., Bugaiska et al., 2019). In our studies, the monitoring of the animate targets during the PM task might have benefited from this automatic-attention capture; that is, their detection would be facilitated as compared with the inanimate items, promoting more correct PM responses.

Following this latter account, one could also speculate about possible effects of animacy on the baseline and filler trials performance. In particular, the automatic attention captured by animates could impair performance in these trials as compared with the inanimate ones. Such a prediction was confirmed only in our Study 2, whereas no effect of animacy was observed in neither Study 1a or 1b. Moreover, the response time data has also been used as an indicator of the attentional mechanisms associated with animacy. For example, the response times in a color-naming Stroop task were longer when words refered to animates than to inanimates (e.g., Bugaiska et al., 2019). In our case, no effect of animacy was found on the response times of the baseline and filler trials (see Supplemental Materials). In sum, the predictions based on this account are not consistent with our results (see also Rawlinson & Kelley, 2021), revealing that the animacy effect in PM may not be explained solely by the attention-prioritization account. Other studies manipulating the characteristics of the target and the baseline/filler words, for example, in terms of emotionality, have found similar results: an enhancement of the PM performance for the emotional (target) words, as compared with the neutral ones, but no difference between them on the ongoing task (filler trials; May et al., 2012).

All in all, the present work reinforces the importance of animacy in memory functioning and adds PM to the list of processes that benefit from animacy. Additionally, not considering such variable might lead to disparate results. For example, emotionally valenced items are more likely to involve animates than neutral ones (e.g., May et al., 2012, 2015). Prospective memory research has also used materials that are ambiguous with respect to animacy (Félix et al., 2023; Lowder & Gordon, 2015). These include categories such as fruits, plants, body parts, and natural forces (e.g., Guynn, 2003; Moyes et al., 2019; Thomas & McBride, 2016). At this point, we cannot inform if and how this animacy category affects PM. Finally, we would encourage researchers to consider the variable of animacy when selecting their research materials, as is usually done for other variables (e.g., arousal, word frequency; May et al., 2012). Recent work has reported some differences on animacy ratings depending on the participants’ language and age; thus, specific language and age-group norms should be used (Félix et al., 2023).

Besides the theoretical relevance of the animacy effect in PM, one can speculate about the potential interest of these results to more applied contexts. Considering that PM is crucial to maintain a functional and independent life, one needs (and uses) PM ubiquitously. At the same time, most of our daily memory failures are PM-related (Cockburn, 1995), which can have severe consequences (e.g., Dembitzer & Lai, 2003). Thus, it is crucial to find the best tools to improve PM functioning. Our results suggest that using the naturally existing mnemonic tuning toward animates might be one such tool.

### Supplementary information

ESM 1 (PDF 298 kb)

## Data Availability

The datasets generated during and/or analyzed during the current study are available in the OSF repository (https://osf.io/g6uqt/?view_only=None).

## Footnotes

1 For these analyses, participants performing at 100% on the PM task were excluded, as no room existed for a possible effect to occur (59 participants in Study 1a, 20 in Study 1b, and 45 in Study 2).
