# Supplementary material for: The breadth of animacy in memory: New evidence from prospective memory
Source: Psychon Bull Rev. 2023 Nov 27;31(3):1323–34. doi: 10.3758/s13423-023-02406-y (PMC11192816; doi:10.3758/s13423-023-02406-y)
Supplement: Supplementary file 1 — (PDF 298 kb) [file 13423_2023_2406_MOESM1_ESM.pdf]

The Breadth of Animacy in Memory: New Evidence from Prospective Memory

Supplemental Material

Words Used in Studies 1a, 1b and 2

Selected Words for Study 1a and Study 2

	Animate words	Inanimate words
**Practice phase**	COUSIN	SHIRT
**Baseline phase**	BOY	BUILDING
	DOCTOR	CROWN
	DOVE	JUG
	HORSE	KEY
	KING	PENCIL
	LION	PILLOW
	RABBIT	PLANE
	WIFE	UMBRELLA
**PM phase: Filler words**	BROTHER	BOWL
	CAT	CELLAR
	FROG	COIN
	HAWK	DRESS
	LAMB	FLAG
	MONKEY	FORK
	OWL	GOLD
	PRIEST	JACKET
	QUEEN	KETTLE
	RAT	LAMP
	TEACHER	STOVE
	WOMAN	TRUCK
**PM phase: Target words**		
**Version DB**	DANCER	BOTTLE
**Version NP**	NURSE	PHONE

Selected Words for Study 1b

	Animate words		Inanimate words	
	**Portuguese word**	**English translation**	**Portuguese word**	**English translation**
**Practice phase**	*BORBOLETA*	BUTTERFLY	*CANDEEIRO*	LAMP
**Baseline phase**	*COELHO*	RABBIT	*BARRIL*	BARREL
	DENTISTA	DENTIST	*BEBIDA*	DRINK
	DOUTOR	DOCTOR	*CARTA*	LETTER
	FALCÃO	HAWK	*CHAPÉU*	HAT
	IRMÃO	BROTHER	*LAÇO*	RIBBON
	PADRE	PRIEST	*MESA*	TABLE
	RAPAZ	BOY	*PAPEL*	PAPER
	SAPO	FROG	*REBUÇADO*	CANDY
**PM phase: Filler words**	*ADULTO*	ADULT	*BANCO*	BANK
	ATOR	ACTOR	*CADEIRA*	CHAIR
	CÃO	DOG	*CESTO*	BASKET
	CORUJA	OWL	*CHAVE*	KEY
	CRIANÇA	KID	*DIAMANTE*	DIAMOND
	ESCRITOR	WRITER	*GARRAFA*	BOTTLE
	GALINHA	CHICKEN	*LÁPIS*	PENCIL
	GATO	CAT	*MARTELO*	HAMMER
	PÁSSARO	BIRD	*OURO*	GOLD
	POMBA	DOVE	*TARTE*	PIE
	PORCO	PIG	*TESOURA*	SCISSORS
	VACA	COW	*VESTIDO*	DRESS
**PM phase: Target words**				
**Version CJ**	*CAVALO*	HORSE	*JANELA*	WINDOW
**Version AC**	*ATLETA*	ATHLETE	*CAMISA*	SHIRT

Details about the Procedure, Data Analyses and Fine-Grained Results

Additional Information about Excluded Participants

The PM literature is not consistent regarding the inclusion/exclusion of participants who did not provide any correct PM response (e.g., Horn & Bayen, 2015, included those participants, while Gilbert, 2015, excluded them). In our study, as data were collected online, we opted to exclude those participants because missing PM responses could be due to a normal PM failure, or to other non-controlled factors (e.g., misreading instructions, PM responses not being registered due to nonstandard keyboard layouts, cf. Gilbert, 2015–supplementary information). However, for each study, we conducted an additional 2 (Animacy: animates vs. inanimates) x 3 (Type of trial: baseline vs. filler vs. target) repeated measures ANOVA, including also the participants who were excluded for not performing any PM response. Across studies, the results revealed the same pattern as when excluding them (Supplementary Table S1), including the follow-up paired t-tests used to disentangle the interactions.

Supplementary Table S1.

Statistical Analysis for Performance Including the Participants Who Did Not Perform Any PM Response in Each Study

Study	Animacy	Type of trial	Animacy X Type of Trial	Follow-up paired t-tests
Study 1a	$F(1, 229) = 7.26$,	$F(1.17, 268.50) = 120.88$,	$F(1.32, 301.22) = 19.68$,	**B**: $t(229) = -2.34$, $p = .020$
($N = 230$)	$p = .008$, $\eta_p^2 = .031$	$p < .001$, $\eta_p^2 = .345$	$p < .001$, $\eta_p^2 = .079$	**F**: $t(229) = -1.48$, $p = .139$
				**T**: $t(229) = 4.34$, $p < .001$, $dz = 0.29$
Study 1b	$F(1, 114) = 6.15$,	$F(1.15, 130.71) = 71.81$,	$F(1.51, 172.46) = 6.42$,	**B**: $t(114) = 0.58$, $p = .562$
($N = 115$)	$p = .015$, $\eta_p^2 = .051$	$p < .001$, $\eta_p^2 = .386$	$p = .002$, $\eta_p^2 = .053$	**F**: $t(114) = -1.27$, $p = .206$
				**T**: $t(114) = 2.88$, $p = .005$, $dz = 0.27$
Study 2	$F(1, 93) = 2.16$,	$F(1.10, 102.07) = 39.87$,	$F(1.23, 114.31) = 13.04$,	**B**: $t(93) = -3.68$, $p < .001$, $dz = -0.38$
($N = 94$)	$p = .145$	$p < .001$, $\eta_p^2 = .300$	$p < .001$, $\eta_p^2 = .123$	**F**: $t(93) = -1.36$, $p = .178$
				**T**: $t(93) = 3.02$, $p = .003$, $dz = 0.31$

Notes: N = Sample size after including the participants who did not give any correct PM response. The significance of the follow-up comparisons should be interpreted considering the value $p < .0167$ resultant from the Bonferroni correction for the multiple comparisons. “B”, “F” and “T” stand for “Baseline trials”, “Filler trials” and “Target trials”, respectively.

Exclusion of Trials from the Analysis of Performance

Following previous studies (e.g., Smith & Hunt, 2014), trials immediately after the target trials were excluded, as performance on these trials may incur an additional cost due to the PM response. Trials with missing responses were also excluded from the analyses (as in, for example, Strickland et al., 2020). In **Study 1a**, a total of 0.7% of the baseline, 2.0% of the filler, and 1.0% of the target trials were excluded from the analyses for those reasons. In **Study 1b**, these corresponded to 2.2% of the baseline, 3.3% of the filler, and 2.6% of the target trials. In **Study 2**, 1.4% of the baseline, 0.9% of the filler, and 1.6% of the target trials were excluded for the same motives.

Versions of the Task

In Studies 1a and 2, versions 1DB and 2DB presented the target words “bottle” and “dancer”, whereas versions 1NP and 2NP presented “nurse” and “phone” as targets. In Study 1b, versions 1CJ and 2CJ presented the target words *“cavalo”* [horse] and *“janela”* [window], whereas *“atleta”* [athlete] and *“camisa”* [shirt] were presented in versions 1AC and 2AC. In the PM phase, words were presented in a fixed order to every participant but counterbalanced between versions: where an animate word was presented in Version 1, an inanimate was presented in Version 2 (and vice-versa). For each study, participants were allocated to the experimental versions as follows: **Study 1a**: Version 1DB (n = 40), 2DB (n = 46), 1NP (n = 46), 2NP (n = 44); **Study 1b**: 1CJ (n = 19), 2CJ (n = 22), 1AC (n = 20), 2AC (n = 17); and **Study 2**: 1DB (n = 19), 2DB (n = 22), 1NP (n = 17), 2NP (n = 21).

Due to the slightly unbalanced number of participants allocated to each version, we explored if the variable Version of the experiment influenced our results in any way, by conducting a 2 (Animacy: animates vs. inanimates) x 3 (Type of trials: baseline vs. filler vs. target) x 4 (Version) mixed ANOVA, for each study. Across studies, neither the main effect

of Version of the experiment, nor the interactions involving this variable, were significant (**Study 1a**: lowest p = .505; **Study 1b**: lowest p = .147; **Study 2**: lowest p = .114). This suggests that the animacy effects reported in all studies are not restricted to one specific animate / inanimate PM target, nor to a specific order of presentation of the PM targets (i.e., being presented first with an animate or an inanimate target did not influence the overall PM performance).

False Alarms

The false alarm response rate (i.e., giving a PM response to a filler trial) was negligible and did no differences between animates and inanimates were obtained across studies, as presented in Supplementary Table S2.

Supplementary Table S2.

Average Number of False Alarm Responses Given to Animate and Inanimate Trials, and Corresponding Comparisons (Paired T-Tests), for Each Study

Study	Animates (*SD*)	Inanimates (*SD*)	Paired t-tests
Study 1a	0.04 (0.20)	0.03 (0.17)	$t(175) = 0.63$, $p = .529$
Study 1b	0.03 (0.16)	0.01 (0.11)	$t(77) = 0.58$, $p = .567$
Study 2	0.03 (0.16)	0.03 (0.16)	$t(78) < 0.01$, $p > .999$

Analyses of the Response Times (RTs)

The baseline phase provides a “purer” measure of the participants’ performance in the ongoing task only, allowing us to explore the cost/interference of performing the ongoing task with an embedded PM task (PM phase). Such cost/interference is usually indexed by the participants’ response times. As data were collected online, analyses of response times assumed a secondary role in this work, as these data might vary due to several uncontrollable factors (e.g., the participants’ internet speed).

Only the RTs from correctly-responded trials were considered (i.e., press Y, N or SPACEBAR in color-match, color-nonmatch and target trials, respectively). In these analyses, we only included data from the participants who provided correct responses to both animate and inanimate stimuli across all types of trials. Missing-response trials were not considered in these analyses (as in Strickland et al., 2020). Following previous studies, RTs were trimmed separately for animate and inanimate trials (e.g., Rummell et al., 2017 used a lexical decision task, and trimmed RTs separately for words and nonwords); those trials with RTs below M-3SD or above M+3SD from each participant’s mean were excluded (Matos et al., 2020; as similarly done by Smith and Hunt, 2014). RTs from target trials were not trimmed, otherwise there would be too few trials/datapoints to analyze. This trimming procedure resulted in the exclusion of 0.1% of the total trials in **Study 1a**, 0.1% of the baseline and 0.4% of the filler trials in **Study 1b**, and 1.0% of the total trials in **Study 2**. Results are depicted in Supplementary Fig. S1, and the statistical analyses are presented in Supplementary Table S3.

Supplementary Fig. S1.

Response Times Obtained in Studies 1a (N = 132), 1b (N = 63) and 2 (N = 72). Error Bars Represent Standard Errors of the Mean

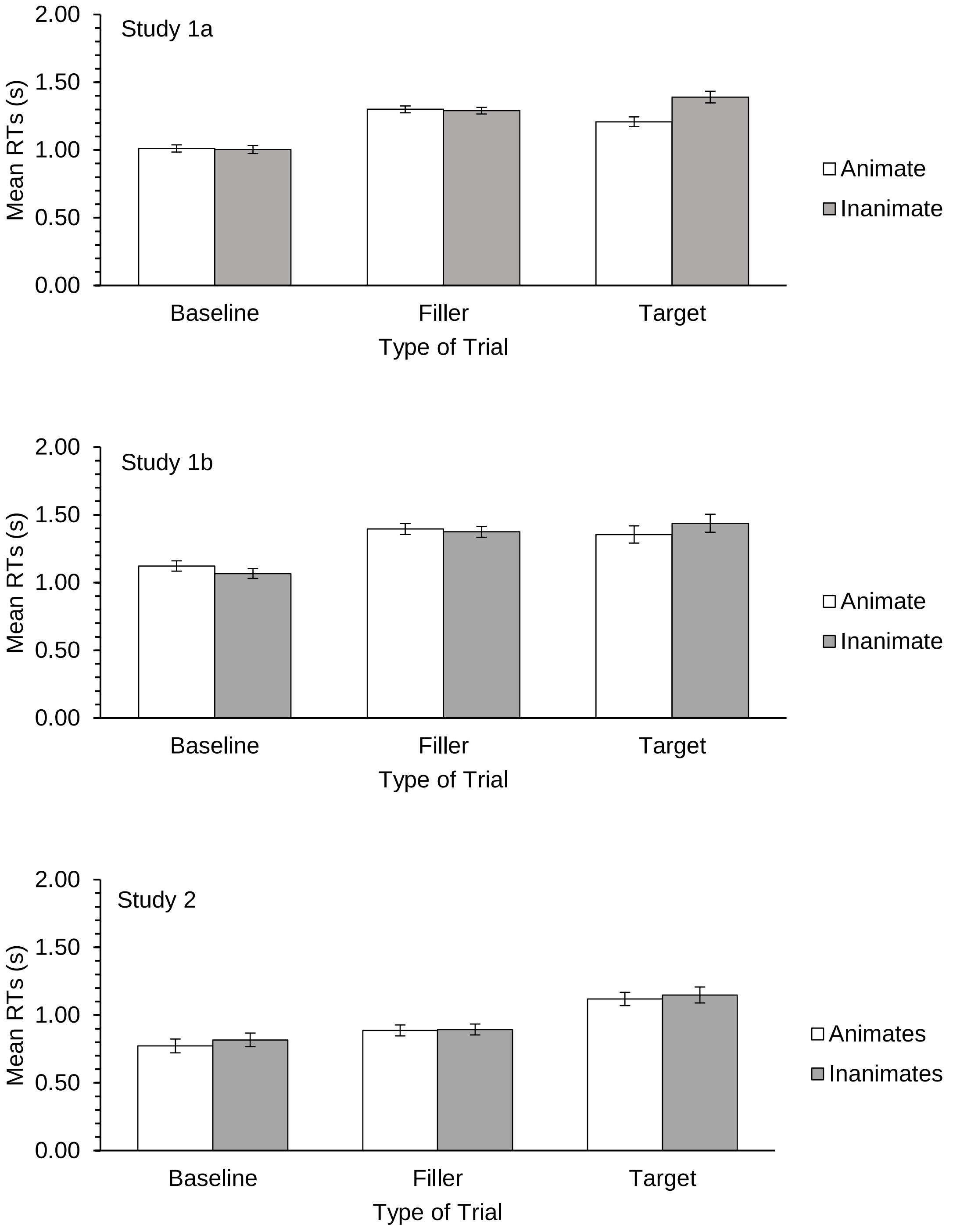

Supplementary Table S3.

Statistical Analyses on the Response Times for Each Study

Study	Animacy	Type of Trial		Animacy X Type of Trial
	Main effect	Main effect	Follow-up paired t-tests	Interaction
Study 1a (*N* = 132)	**$F(1, 131) = 10.36$, $p = .002$, $\eta_p^2 = .073$**	**$F(1.49, 195.33) = 65.33$, $p < .001$, $\eta_p^2 = .333$**	**B vs. F: $t(131) = -15.13$, $p < .001$, $dz = -1.32$**<br>**B vs. T: $t(131) = -8.11$, $p < .001$, $dz = -0.71$**<br>F vs. T: $t(131) = 0.54$, $p = .59$	**$F(1.29, 169.38) = 12.12$, $p < .001$, $\eta_p^2 = .085$**
Study 1b (*N* = 63)	$F(1, 62) = 0.004$, $p = .948$	**$F(1.51, 93.63) = 30.20$, $p < .001$, $\eta_p^2 = .328$**	**B vs. F: $t(62) = -9.75$, $p < .001$, $dz = -1.23$**<br>**B vs. T: $t(62) = -5.56$, $p < .001$, $dz = -0.70$**<br>F vs. T: $t(62) = 0.06$, $p = .953$	$F(1.30, 80.38) = 2.58$, $p = .103$
Study 2 (*N* = 72)	$F(1, 71) = 1.38$, $p = .245$	**$F(1.66, 117.88) = 51.45$, $p < .001$, $\eta_p^2 = .420$**	**B vs. F: $t(71) = -3.21$, $p = .002$, $dz = -0.38$**<br>**B vs. T: $t(71) = -8.19$, $p < .001$, $dz = -0.97$**<br>**F vs. T: $t(71) = -7.92$, $p < .001$, $dz = -0.93$**	$F(1.21, 85.98) = 0.26$, $p = .658$

Notes: *N* = Sample included in the RTs analyses (i.e., participants that provided correct responses to both animate and inanimate stimuli across all types of trials). B = Baseline trials; F = Filler trials; T = Target trials. The significance of the follow-up comparisons should be interpreted considering the value p < .0167 following the Bonferroni correction for the multiple comparisons. Statistically-significant results are presented bolded.

The Animacy X Type of Trial interaction found in Study 1a was explored with paired t-tests. Those revealed faster response times towards the animate (than inanimate) stimuli, but only in target trials, $t(131) = -3.75$, $p < .001$, $dz = -.33$.

The further exploration of the main effect of type of trial revealed, **across studies**, a cost to the ongoing task with an embedded PM task (filler trials), as compared to the baseline trials (i.e., performing the ongoing task only). These results further asseverate the non-focal nature of the task (where a more effortful/strategic retrieving of the targets may occur; Anderson et al., 2019; McDaniel & Einstein, 2000; Smith, 2003). Additionally, in Studies 1a and 1b, responses were also slower in the target trials, as compared to the baseline, but no difference was obtained between the filler and the target trials. This reveals that participants had similar performances in the task, although being conducted in different languages. In Study 2, responses to the target trials were slower than to both the baseline and the filler trials.

Additionally, as presented in Supplementary Fig. S1., RTs from Studies 1a and 1b were very similar. Indeed, both studies followed the same procedure, although in different languages and with different sets of participants. These results further asseverate similar manipulations of the stimuli in both studies, although conducted in different languages. Importantly, the words used in said studies were selected from language-specific animacy word-rating data (Félix et al., 2020; VanArsdall, 2016). Such procedure, which should be followed in future studies, helps to ensure a more adequate manipulation of the variable of animacy. It is worth mentioning the importance of using language-specific animacy ratings in future studies, considering that (despite the high correlation in the mean ratings obtained between different languages) there are some differences in the way words are rated across languages (e.g., Félix et al., 2023).

Performance in the Recognition and Color Naming Tasks

Supplementary Table S4 shows the mean performance in the final recognition and color naming tasks. In the recognition task, participants were presented with words, one at a time, and their task was to decide if those words corresponded to the target trials they were asked to memorize (yes/no forced response). All the targets and four lures (half animates and half inanimates) had been presented during the PM phase.

Supplementary Table S4.

Mean Performance in the Final Target Recognition and Color Naming Tasks in Studies 1a, 1b and 2

Study	Recognition Task		Color naming task
	Targets	Lures	
Study 1a	1.00 (0.04)	0.99 (0.05)	0.98 (0.05)
Study 1b	1.00 (0.00)	0.98 (0.13)	0.98 (0.05)
Study 2	0.97 (0.14)	0.99 (0.08)	--

Note: *SD* is presented in parentheses. The color naming task was implemented only Studies 1a and 1b. The mean performance in the recognition task for Targets corresponds to *hits* and the performance for Lures corresponds to *correct rejections*.

References (Supplemental Materials)

Anderson, F. T., Strube, M. J., & McDaniel, M. A. (2019). Toward a better understanding of costs in prospective memory: A meta-analytic review. *Psychological Bulletin, 145*, 1053–1081. https://doi.org/10.1037/bul0000208

Félix, S. B., Pandeirada, J. N. S., & Nairne, J. S. (2020). Animacy norms for 224 European Portuguese concrete words. *Análise Psicológica, 38*, 257–269. https://doi.org/10.14417/ap.1690

Félix, S. B., Poirier, M., & Pandeirada, J. N. S. (2023). Is "earth" an animate thing? Cross-language and inter-age analyses of animacy word ratings in European Portuguese and British English young and older adults. *PLoS ONE, 18*, e0289755. https://doi.org/https://doi.org/10.1371/journal.pone.0289755

Gilbert, S. J. (2015). Strategic offloading of delayed intentions into the external environment. *The Quarterly Journal of Experimental Psychology*, *68*, 971–992. https://doi.org/10.1080/17470218.2014.972963

Horn, S. S., & Bayen, U. J. (2015). Modeling criterion shifts and target checking in prospective memory monitoring. *Journal of Experimental Psychology: Learning, Memory and Cognition*, *41*, 95–117. https://doi.org/10.1037/a0037676

Matos, P., Santos, F. H., & Albuquerque, P. B. (2020). When we must forget: The effect of cognitive load on prospective memory commission errors. *Memory, 28*, 374–385. https://doi.org/10.1080/09658211.2020.1726399

McDaniel, M. A., & Einstein, G. O. (2000). Strategic and automatic processes in prospective memory retrieval: A multiprocess framework. *Applied Cognitive Psychology, 14*, S127–S144. https://doi.org/10.1002/acp.775

Rummel, J., Smeekens, B. A., & Kane, M. J. (2017). Dealing with prospective memory demands while performing an ongoing task: Shared processing, increased on-task focus, or both? *Journal of Experimental Psychology: Learning, Memory, and Cognition, 43*, 1047–1062. https://doi.org/10.1037/xlm0000359

Smith, R. E. (2003). The cost of remembering to remember in event-based prospective memory: Investigating the capacity demands of delayed intention performance. *Journal of Experimental Psychology: Learning, Memory, and Cognition*, *29*, 347–361. https://doi.org/10.1037/0278-7393.29.3.347

Smith, R. E., & Hunt, R. R. (2014). Prospective memory in young and older adults: The effects of task importance and ongoing task load. *Aging, Neuropsychology, and Cognition*, *21*(4), 411–431. https://doi.org/10.1080/13825585.2013.827150

Strickland, L., Loft, S., & Heathcote, A. (2020). Investigating the effects of ongoing-task bias on prospective memory. *Quarterly Journal of Experimental Psychology*, *73*, 1495–1513. https://doi.org/10.1177/1747021820914915

VanArsdall, J. E. (2016). Exploring animacy as a mnemonic dimension [Doctoral thesis, Purdue University, USA]. https://docs.lib.purdue.edu/open_access_dissertations/873/
